# Shattered Minds: The Growing Psychological Toll of War on Gaza’s Population

**DOI:** 10.1192/j.eurpsy.2026.11513

**Published:** 2026-07-31

**Authors:** M. Ligero Argudo, I. M. Peso Navarro, C. García Cerdán, A. I. Mitadiel Velasco, I. Sánchez Diez, M. Covacho González, A. Macia Casas, E. Domínguez Álvarez

**Affiliations:** 1 Psychiatry; 2 Psychology, Hospital Clínico de Salamanca, Salamanca; 3 Psychiatry, Hospital Provincial de Segovia, Segovia, Spain

## Abstract

**Introduction:**

The prolonged conflict and humanitarian collapse in the Palestinian territory, and specially in the Gaza Strip, have had devastating consequances regarding mental health. Before October 2023, surveys already reported high prevalence of disorders such as post-traumatic stress disorder (PTSD), depression, and anxiety among the population. Since the escalation of the hostilities two years ago, these numbers have increased considerably.

In contrast to the urgent need for psychosocial support, especially among vulnerable populations such as children, women or elders, resources have significally diminished.

**Objectives:**

To synthesize recent evidence on the burdens of mental health problems among the population of Gaza, represent the magnitude of their needs and evaluate recommendations for humanitarian response.

**Methods:**

Narrative review and synthesis of: (1) reports from humanitarian agencies (UNICEF, OCHA, UNRWA, WHO), (2) peer-reviewed and grey literature published between 2023–2025, and (3) thematic analyses from agencies specialized in MHPSS. Priority was given to documents reporting population-level data, mental health surveys, or service assessments on the ground.

**Results:**

- Even though prevalence of specific disorders previous october 2023 is difficult to determine, the WHO stimated that at least 22% of population were living with mental health disorders such as depression, anxiety, PTSD, bipolar disorder or schizophrenia. Since October 2023, humanitarian responders have observed significant increases in PTSD (54% children vs 40% adults), anxiety (34% vs 37%), and depression (41% vs 45%), or psychosomatic problems, among other problems. UNICEF also reports higher substance abuse and addiction. Some reports suggest nearly all children require psychosocial support.
- Mental health system capacity has been nearly vanished: reports show by August 2024 all six mental health centres were closed or forced to evacuate, and the only psychiatric hospital was still non-operational.

**Conclusions:**

Recent evidence reveals a large-scale mental health crisis in Gaza, with universal needs among population and severe structural gaps in care. Urgent priorities include scaling up integrated mental health and psychosocial support in primary care, individual and community-focused programs, rapid training of local providers, and sustained humanitarian access.

Future research should monitor prevalence trends longitudinally and evaluate intervention impact under conflict conditions in preventing chronic and intergenerational trauma.

**Disclosure of Interest:**

None Declared

